# 

**Published:** 1854-07

**Authors:** 


*Appointment.*—Corydon La Ford, M. D., formerly Demonstrator of Anatomy in the University of Buffalo, and more recently Professor of the same branch in the Castleton Medical College, has accepted the chair of Anatomy in the University of Michigan, at Ann Arbor.

Dr. Ford is an accomplished anatomist, and an experienced teacher. His appointment is a judicious one for the interests of the school. H.

